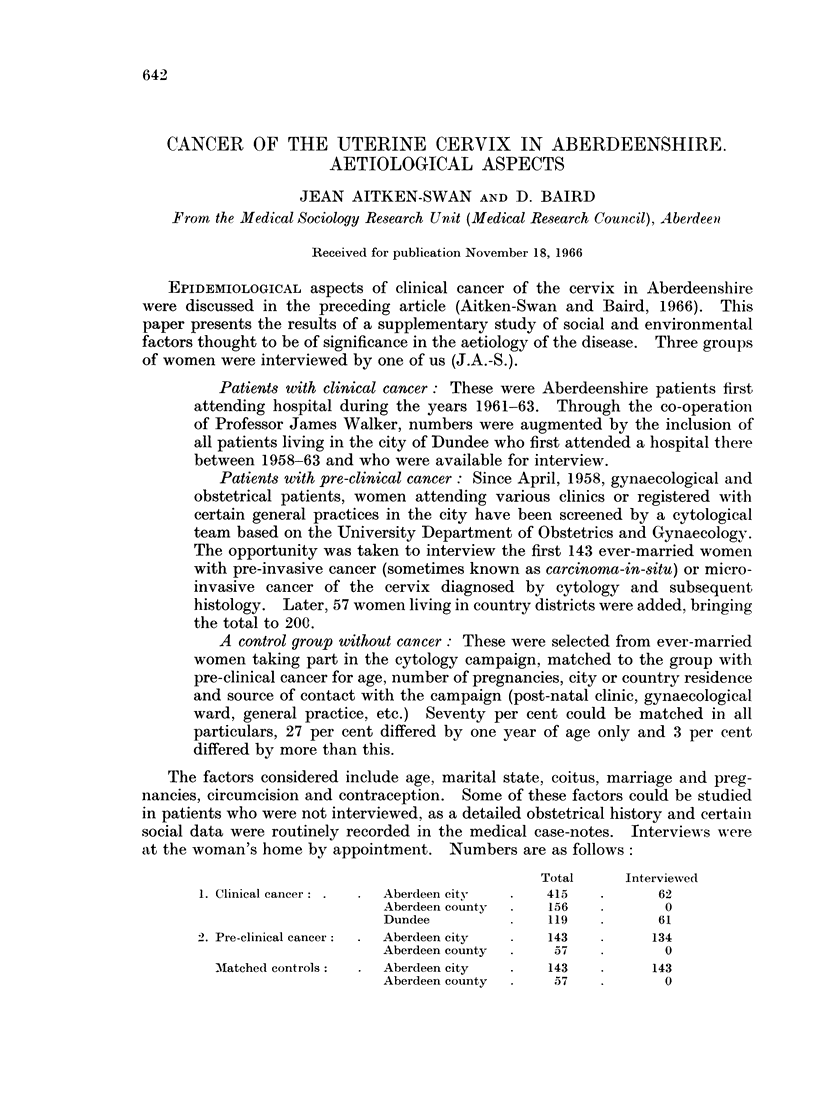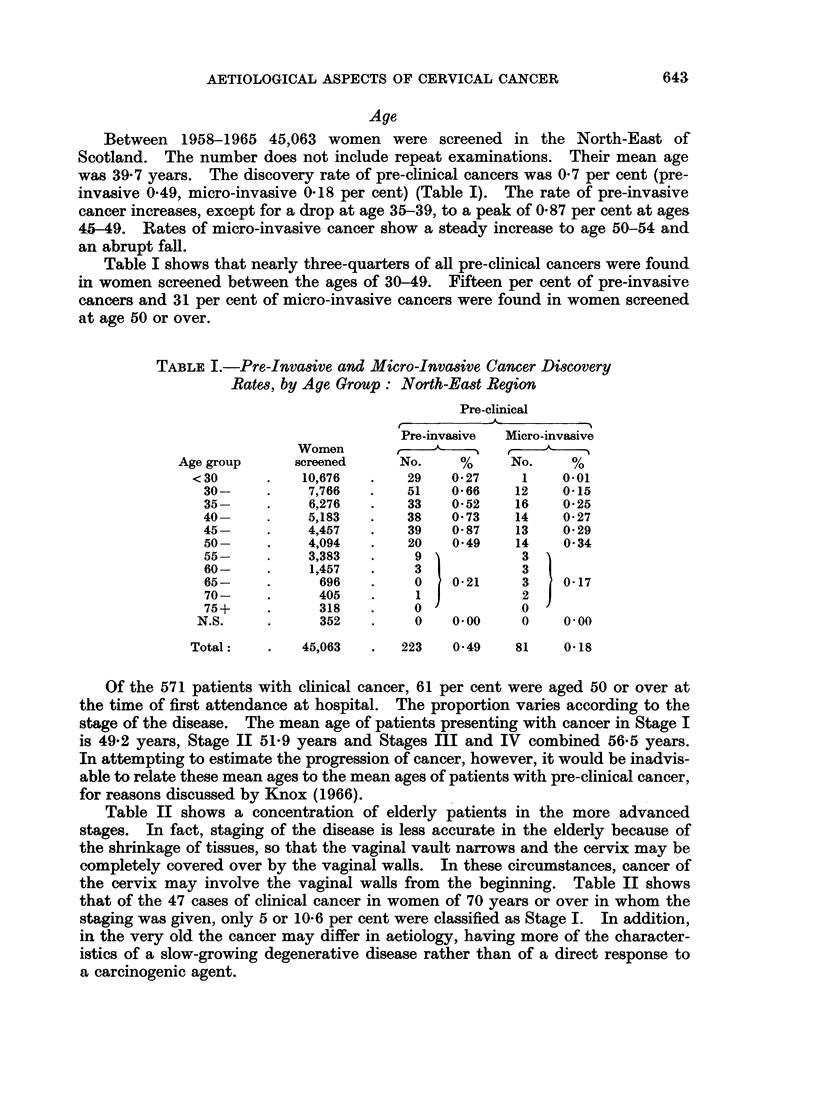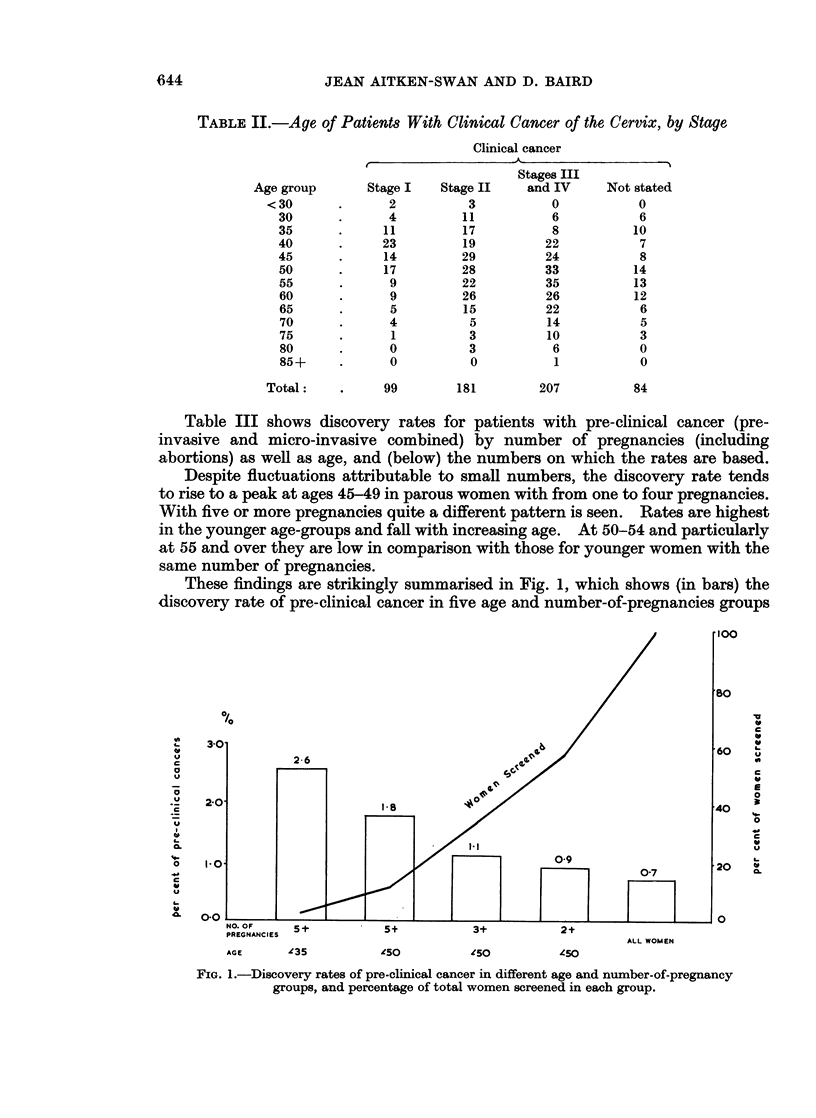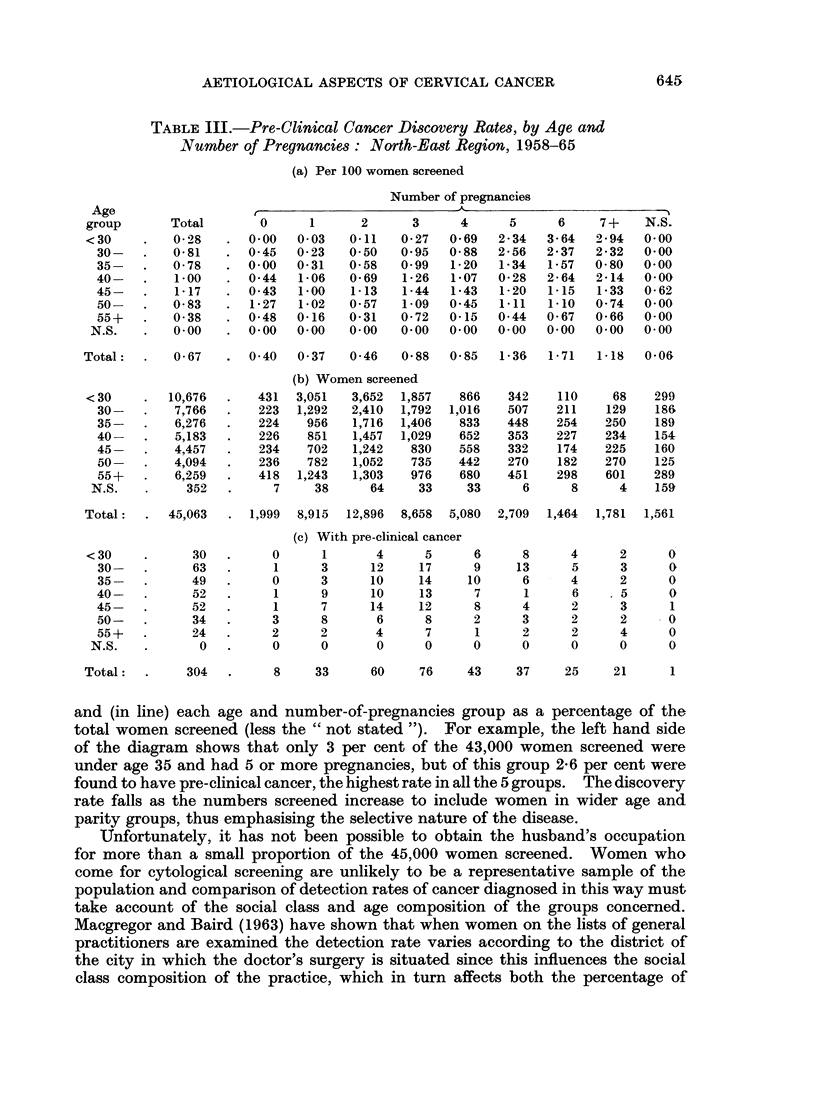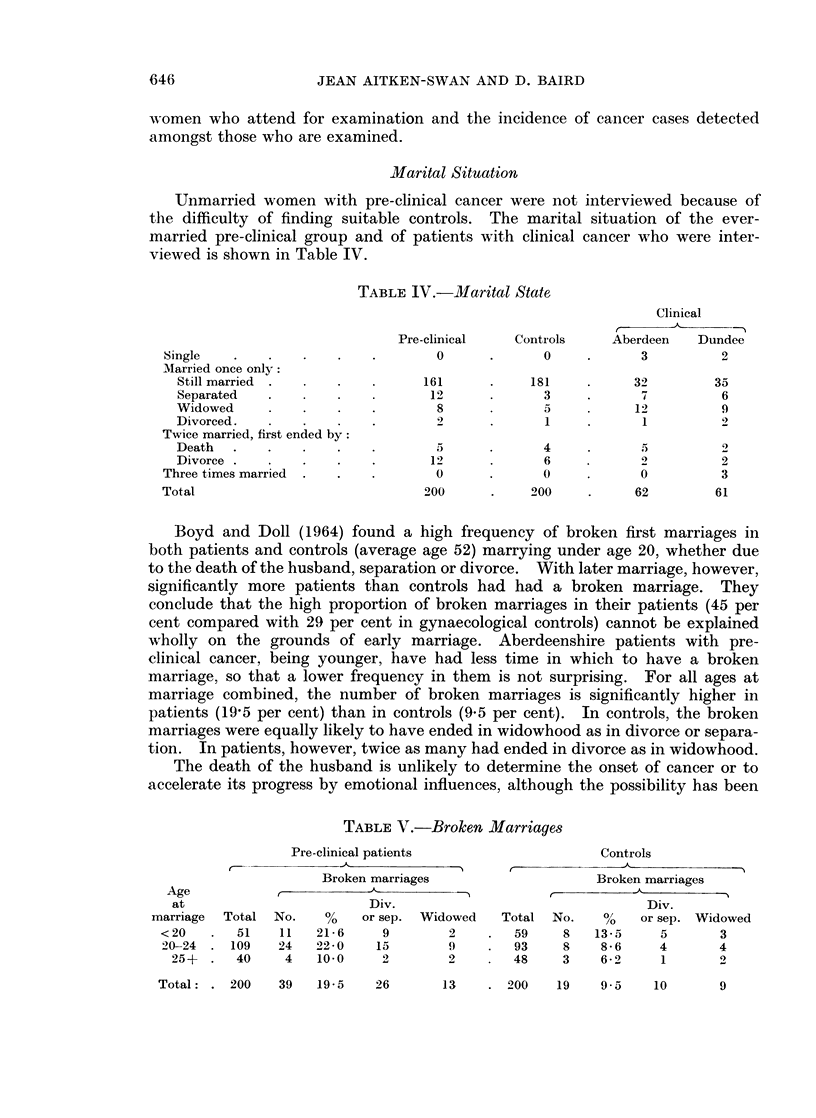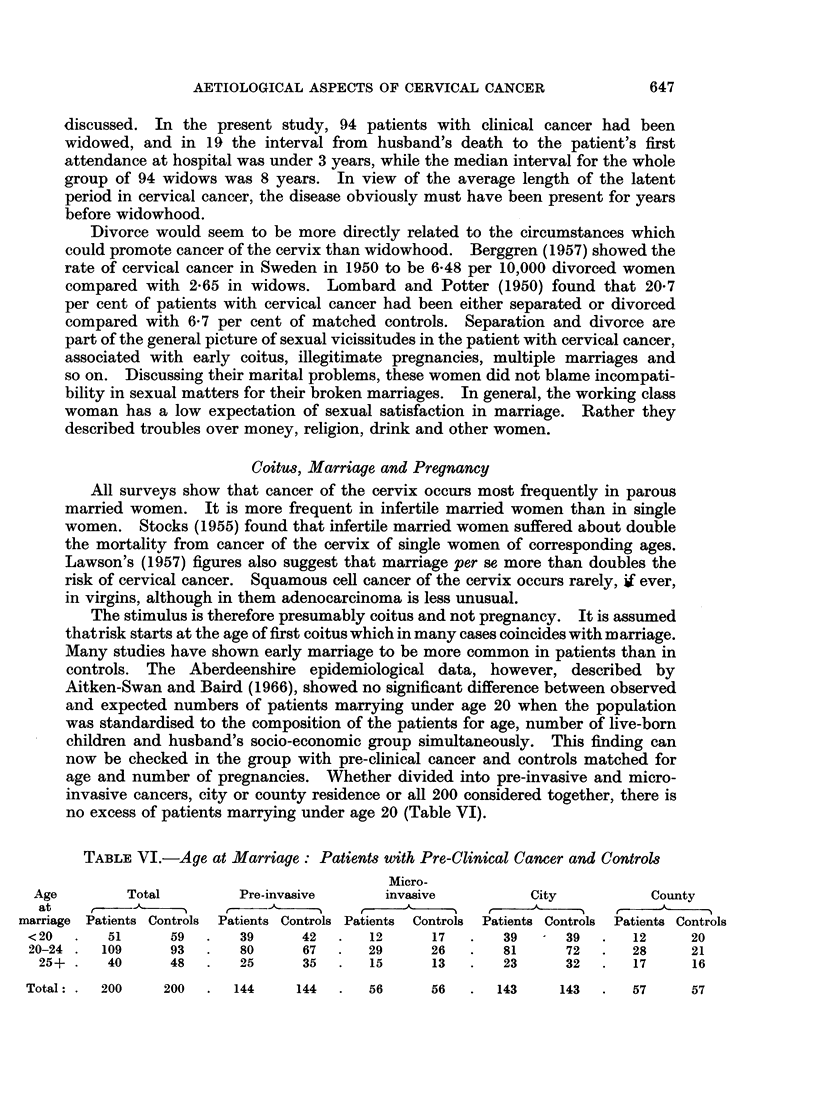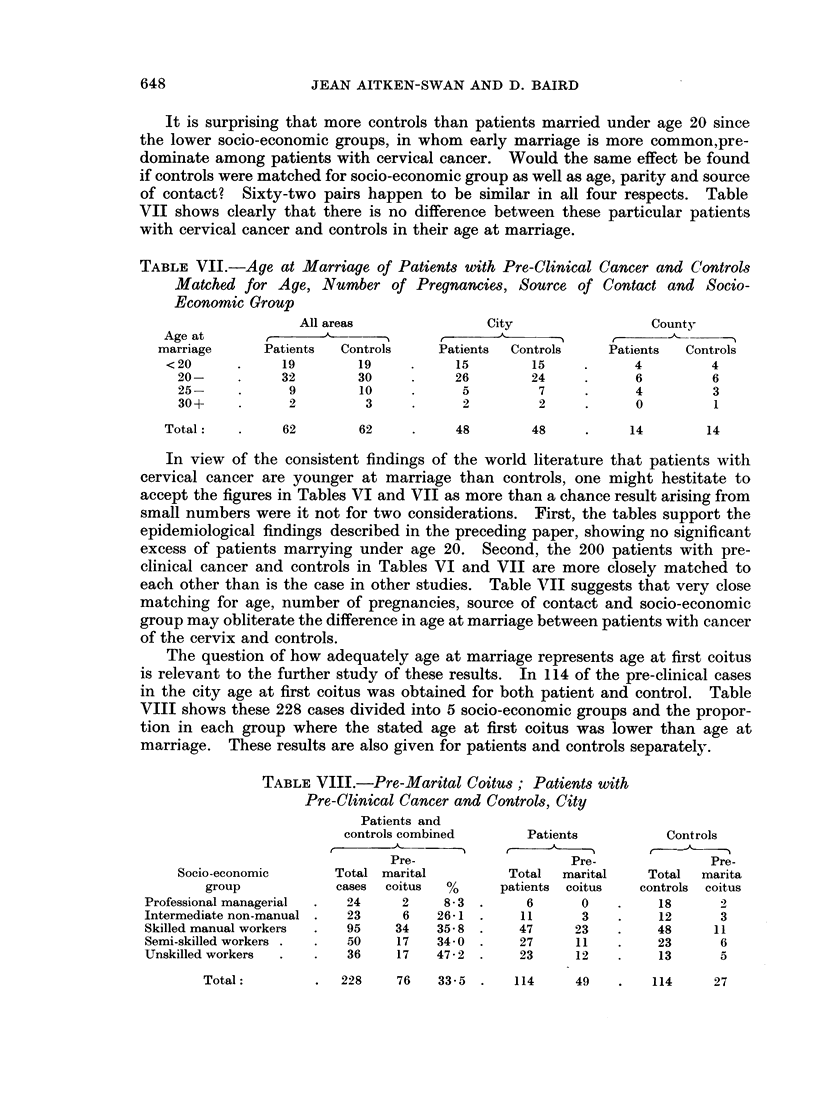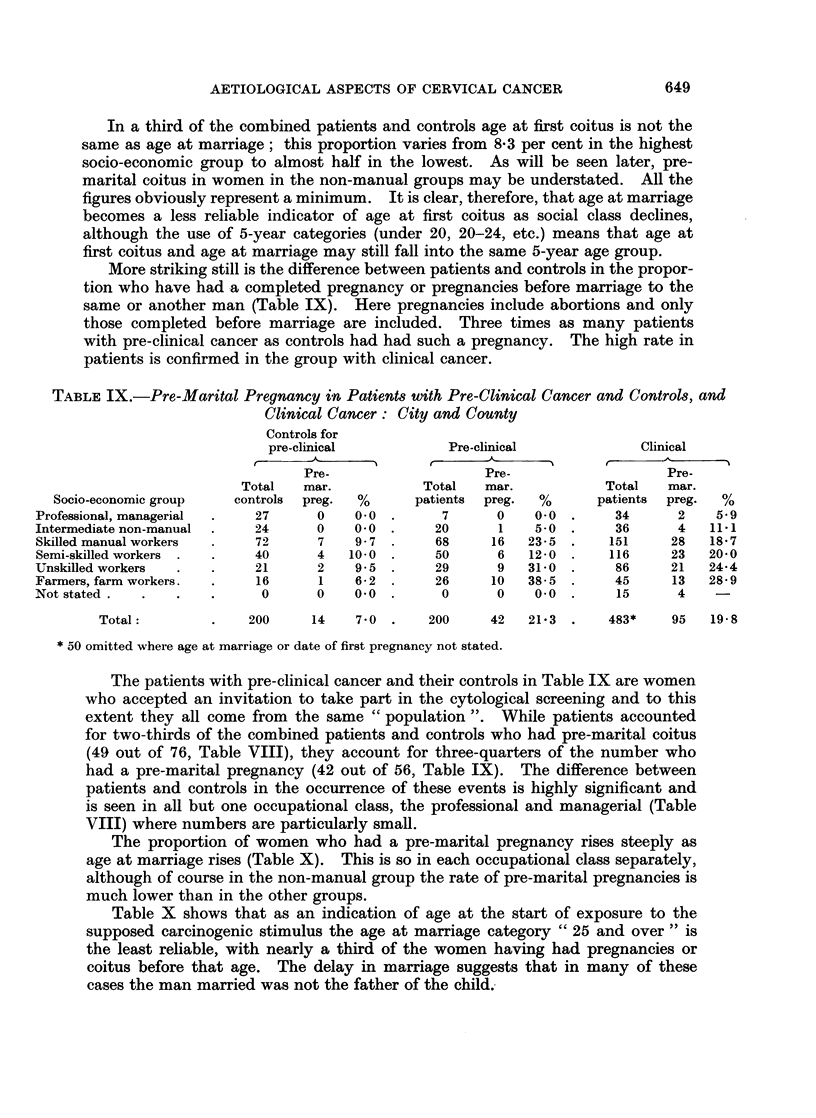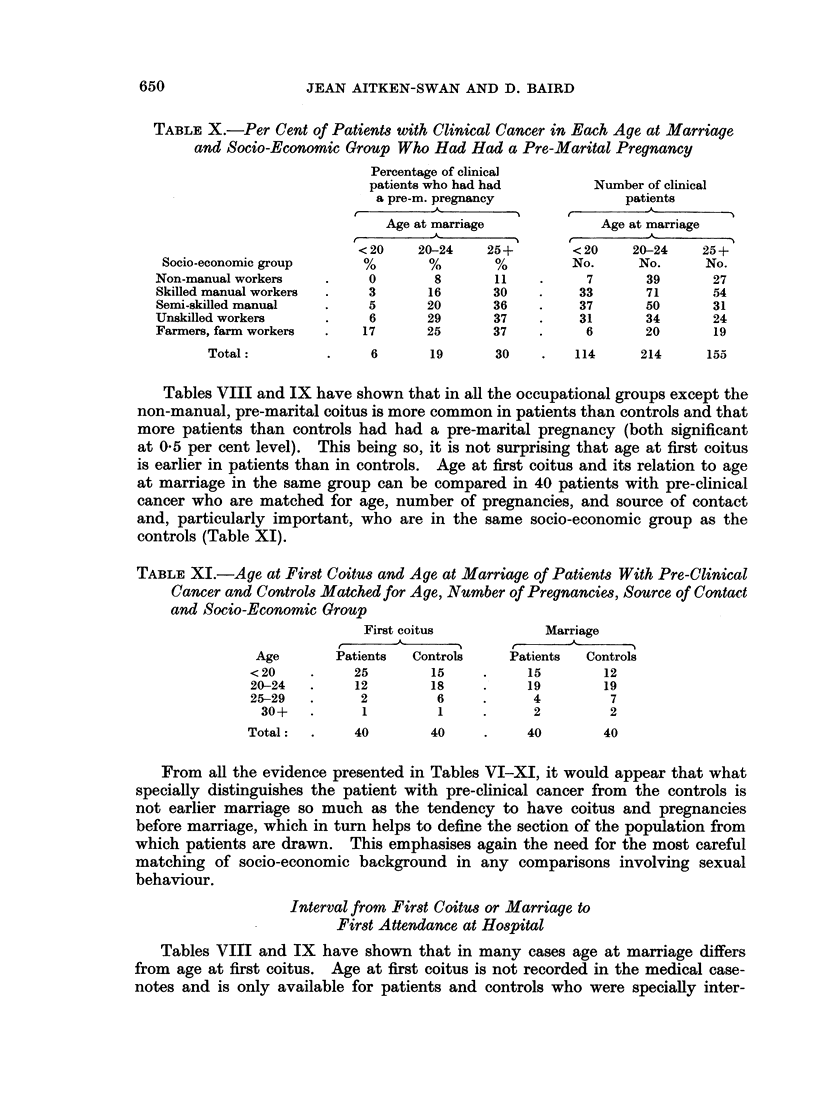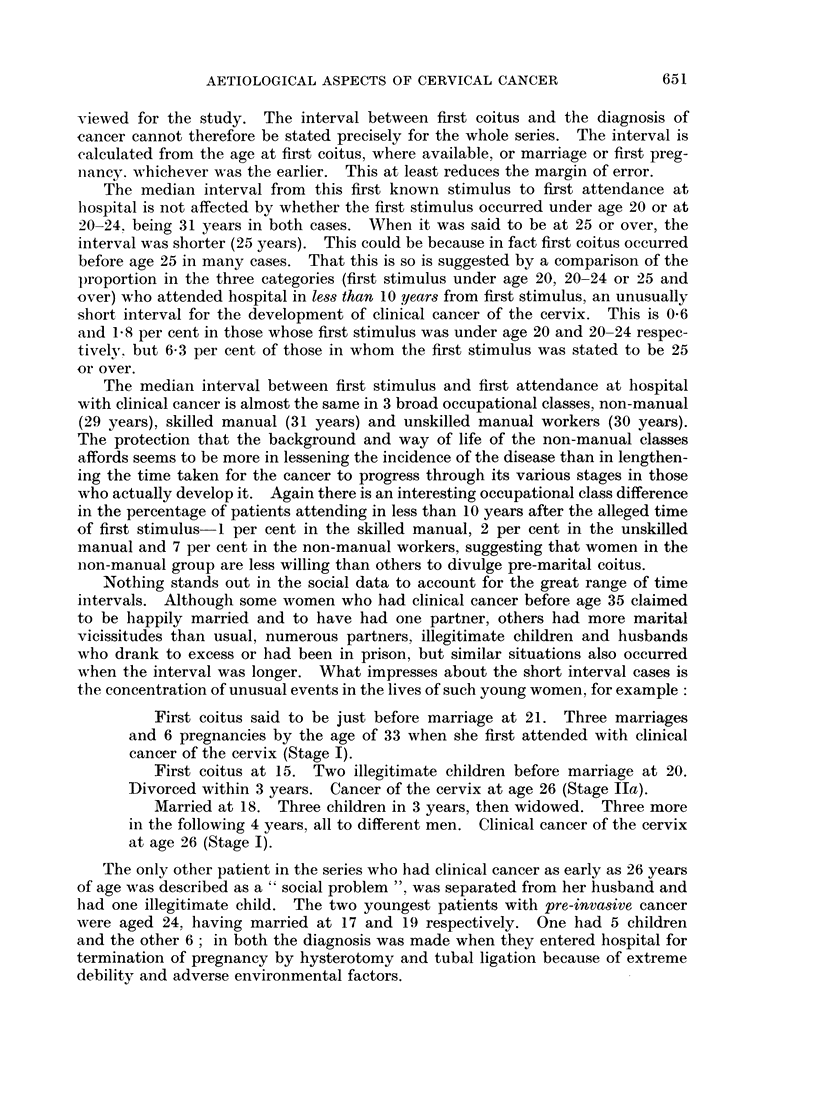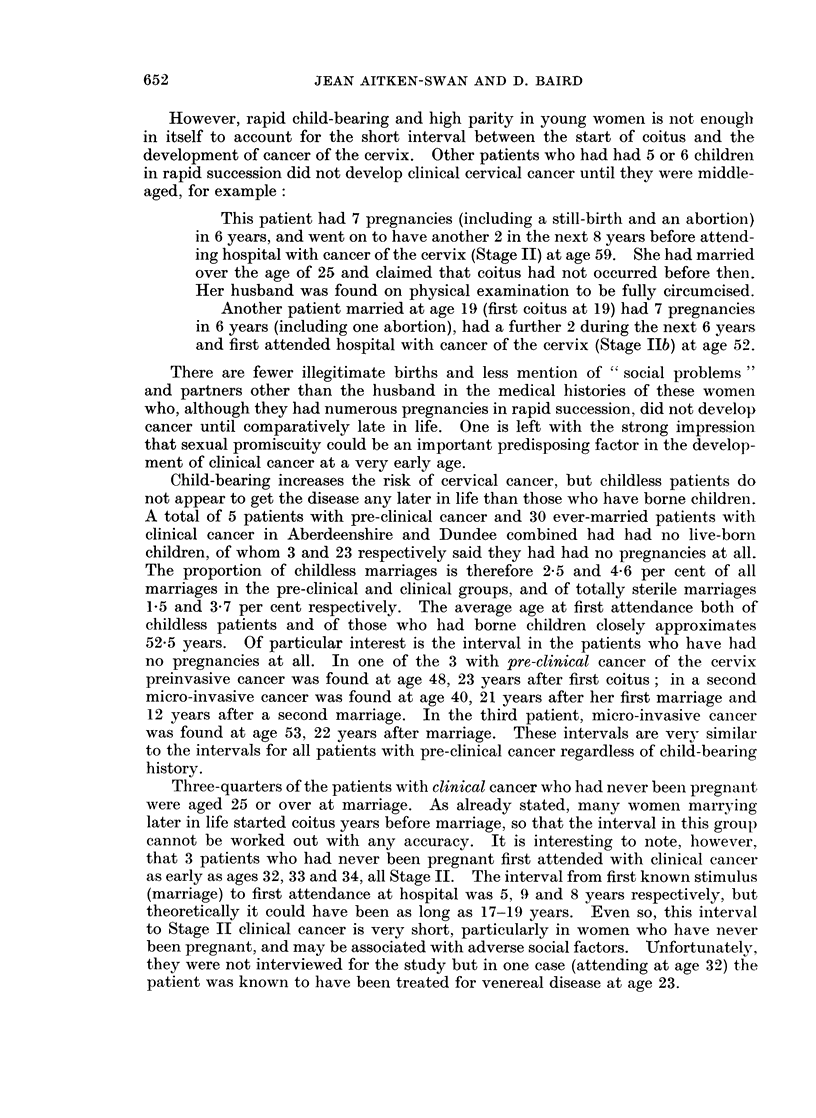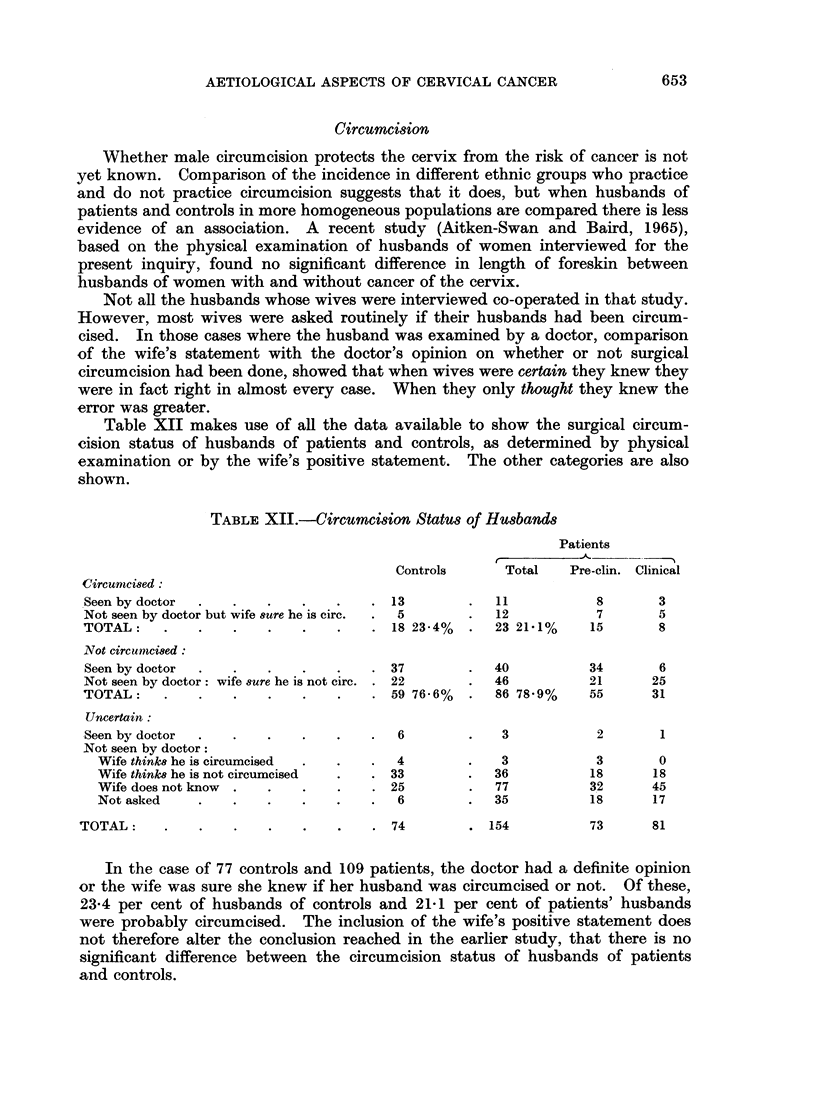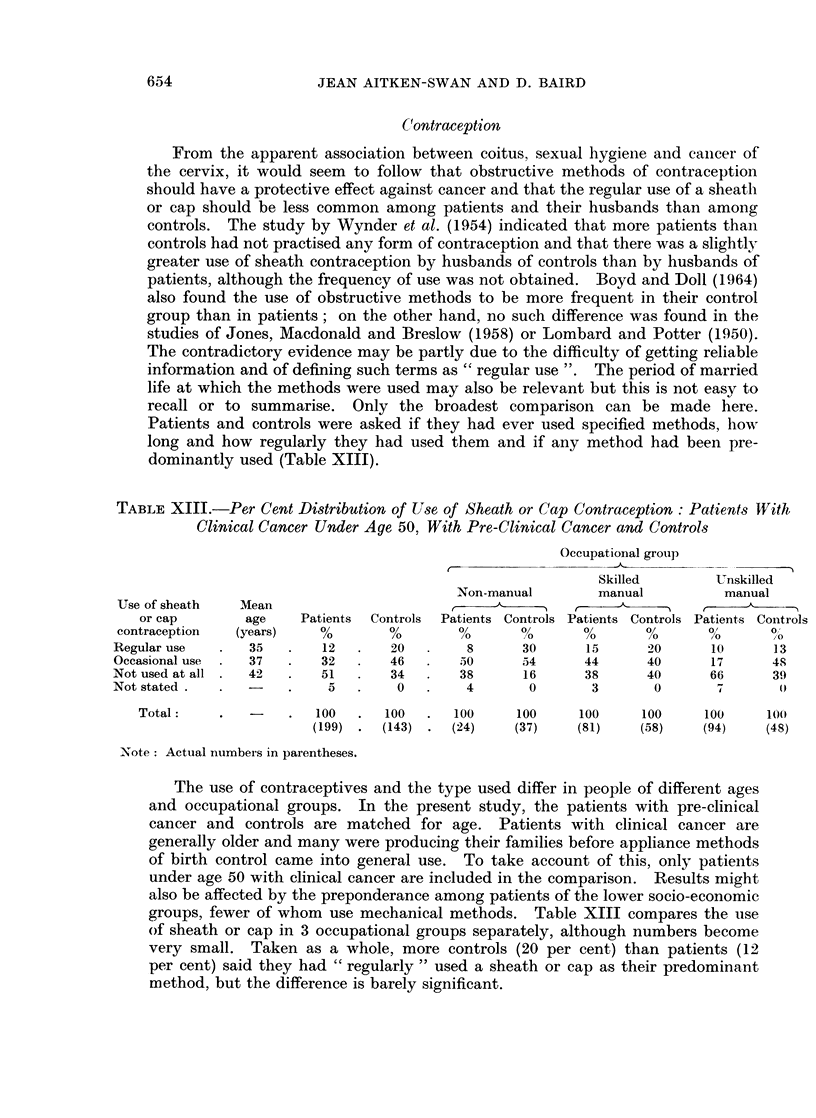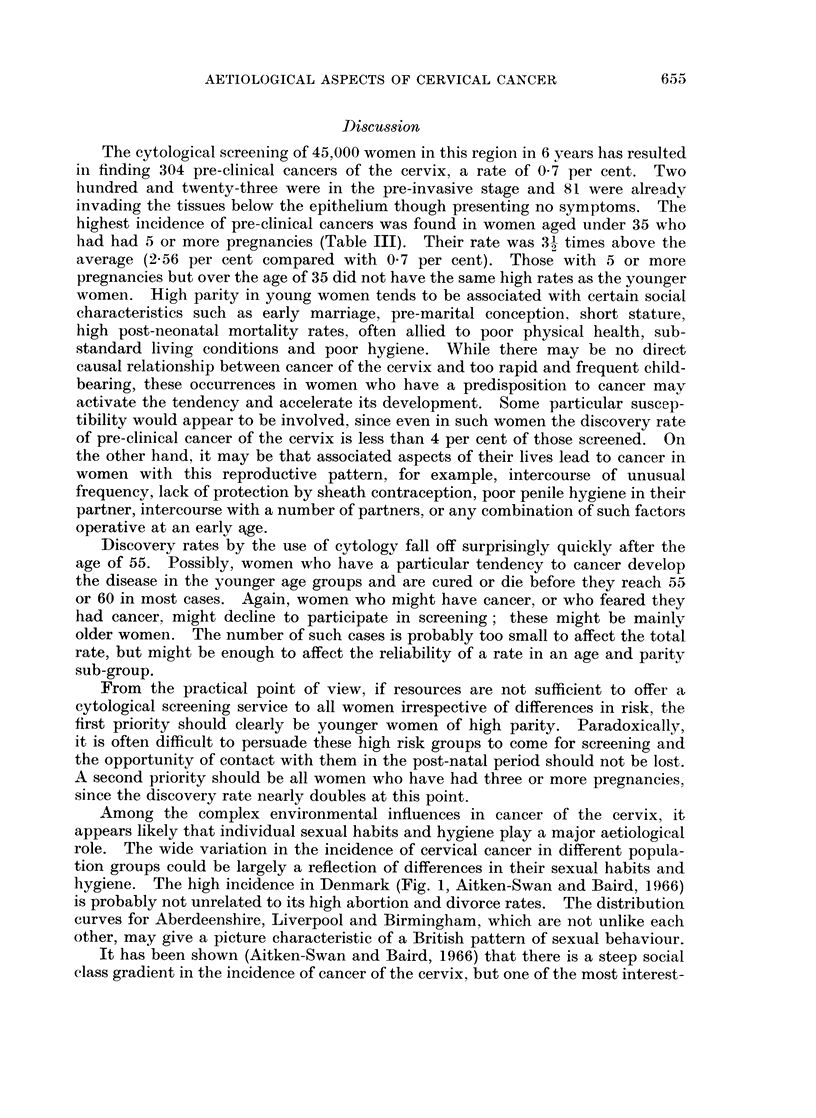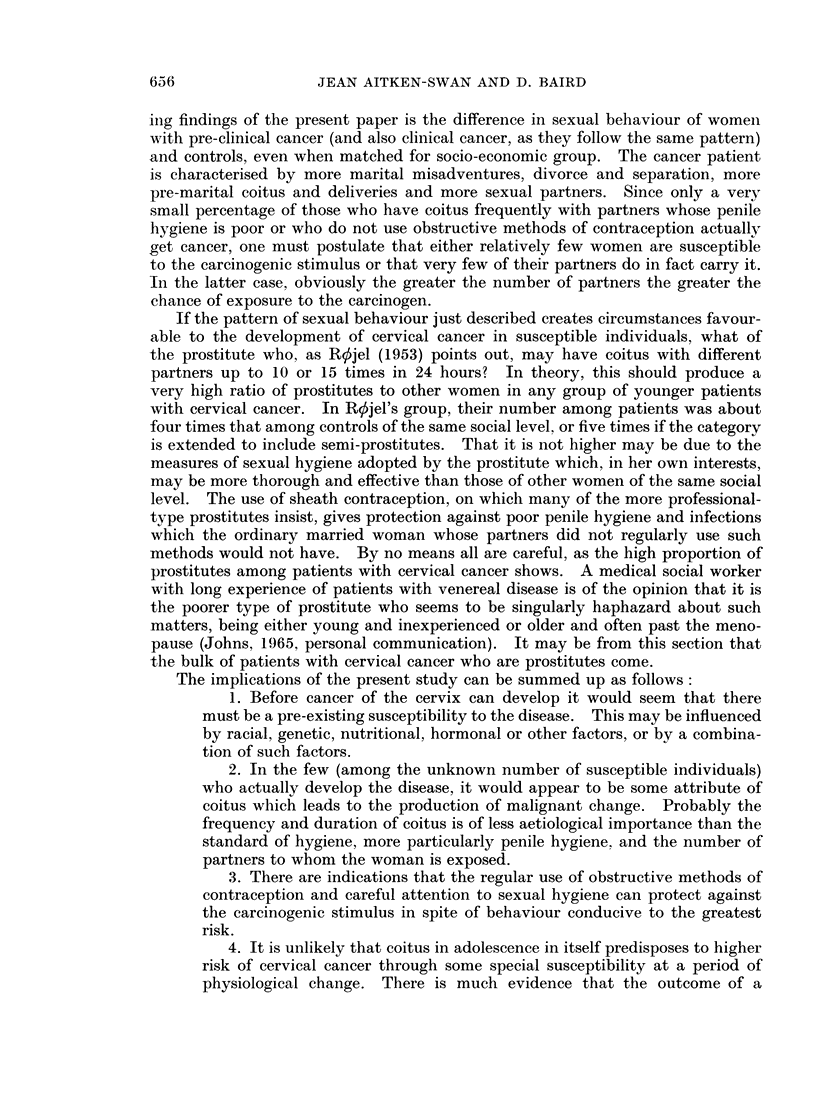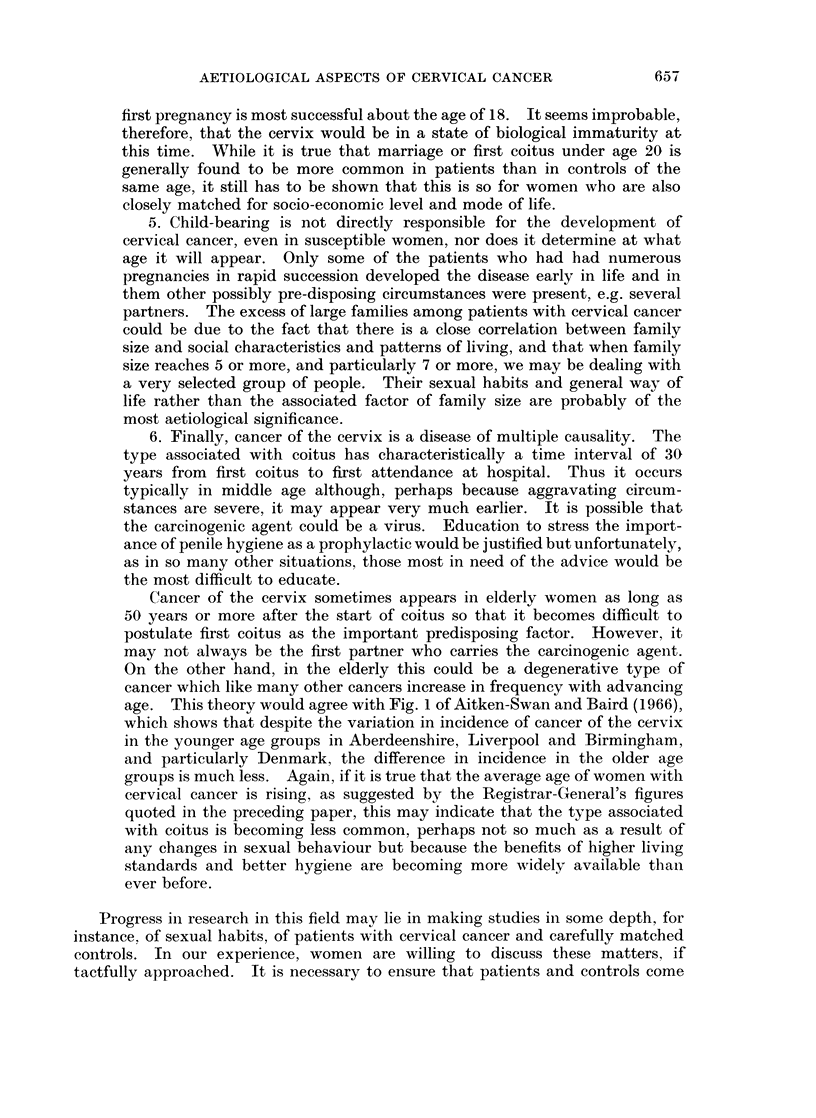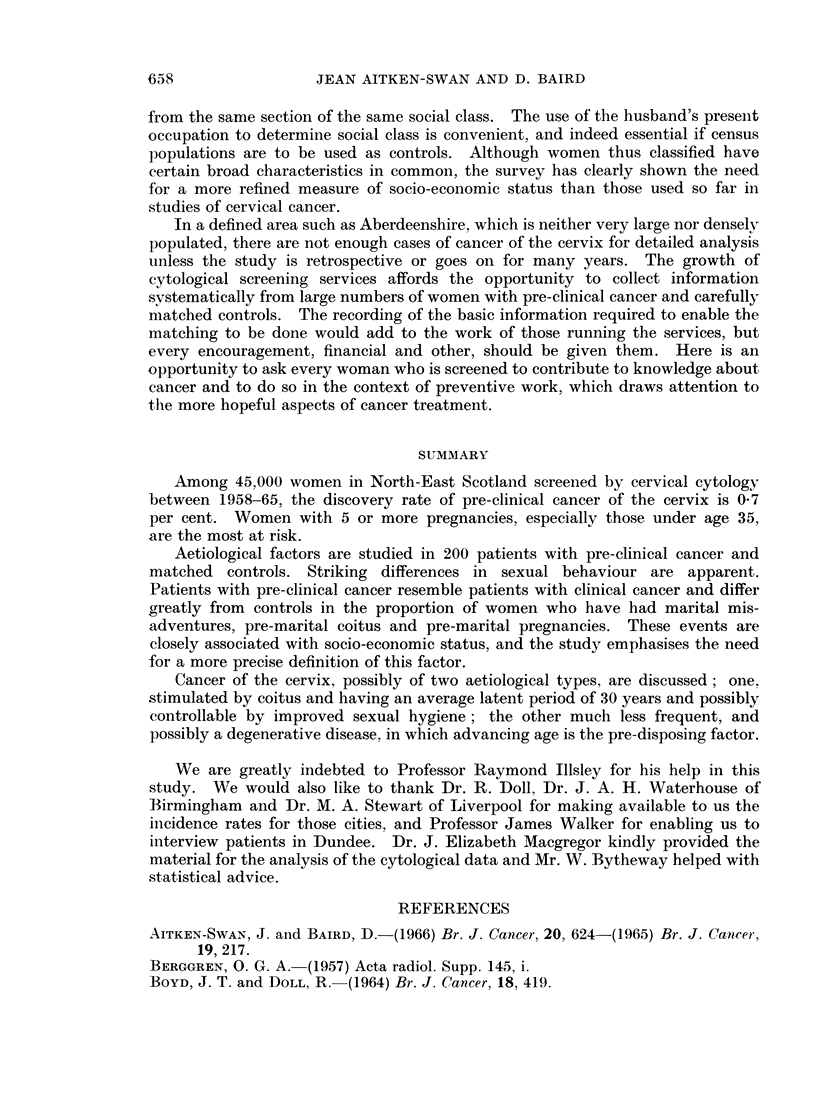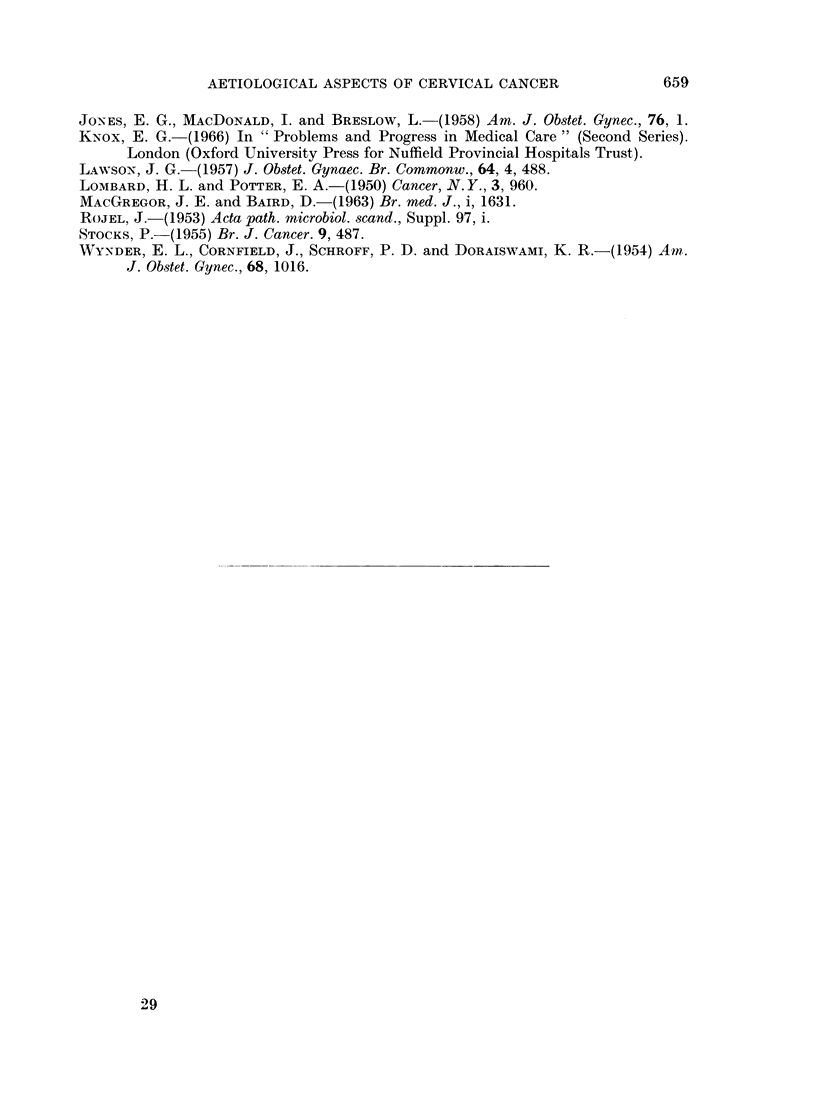# Cancer of the uterine cervix in Aberdeenshire. Aetiological aspects.

**DOI:** 10.1038/bjc.1966.76

**Published:** 1966-12

**Authors:** J. Aitken-Swan, D. Baird


### CANCER OF THE UTERINE CERVIX IN ABERDEENSHIRE. AETIOLOGICAL ASPECTS

JEAN AITKEN-SWAN AND D. BAIRD

*From the Medical Sociology Research Unit (Medical Research Council), Aberdeen*

Received for publication November 18, 1966

EPIDEMIOLOGICAL aspects of clinical cancer of the cervix in Aberdeenshire were discussed in the preceding article (Aitken-Swan and Baird, 1966). This paper presents the results of a supplementary study of social and environmental factors thought to be of significance in the aetiology of the disease. Three groups of women were interviewed by one of us (J.A.-S.).

*Patients with clinical cancer :* These were Aberdeenshire patients first attending hospital during the years 1961–63. Through the co-operation of Professor James Walker, numbers were augmented by the inclusion of all patients living in the city of Dundee who first attended a hospital there between 1958–63 and who were available for interview.

*Patients with pre-clinical cancer :* Since April, 1958, gynaecological and obstetrical patients, women attending various clinics or registered with certain general practices in the city have been screened by a cytological team based on the University Department of Obstetrics and Gynaecology. The opportunity was taken to interview the first 143 ever-married women with pre-invasive cancer (sometimes known as *carcinoma-in-situ*) or micro-invasive cancer of the cervix diagnosed by cytology and subsequent histology. Later, 57 women living in country districts were added, bringing the total to 200.

*A control group without cancer :* These were selected from ever-married women taking part in the cytology campaign, matched to the group with pre-clinical cancer for age, number of pregnancies, city or country residence and source of contact with the campaign (post-natal clinic, gynaecological ward, general practice, etc.) Seventy per cent could be matched in all particulars, 27 per cent differed by one year of age only and 3 per cent differed by more than this.

The factors considered include age, marital state, coitus, marriage and pregnancies, circumcision and contraception. Some of these factors could be studied in patients who were not interviewed, as a detailed obstetrical history and certain social data were routinely recorded in the medical case-notes. Interviews were at the woman's home by appointment. Numbers are as follows :

| | | Total | Interviewed |
|---|---|---|---|
| 1. Clinical cancer : | Aberdeen city | 415 | 62 |
| | Aberdeen county | 156 | 0 |
| | Dundee | 119 | 61 |
| 2. Pre-clinical cancer : | Aberdeen city | 143 | 134 |
| | Aberdeen county | 57 | 0 |
| Matched controls : | Aberdeen city | 143 | 143 |
| | Aberdeen county | 57 | 0 |

### *Age*

Between 1958–1965 45,063 women were screened in the North-East of Scotland. The number does not include repeat examinations. Their mean age was 39·7 years. The discovery rate of pre-clinical cancers was 0·7 per cent (pre-invasive 0·49, micro-invasive 0·18 per cent) (Table I). The rate of pre-invasive cancer increases, except for a drop at age 35–39, to a peak of 0·87 per cent at ages 45–49. Rates of micro-invasive cancer show a steady increase to age 50–54 and an abrupt fall.

Table I shows that nearly three-quarters of all pre-clinical cancers were found in women screened between the ages of 30–49. Fifteen per cent of pre-invasive cancers and 31 per cent of micro-invasive cancers were found in women screened at age 50 or over.

TABLE I.—*Pre-Invasive and Micro-Invasive Cancer Discovery Rates, by Age Group : North-East Region*

| | | Pre-clinical | | | |
|---|---|---|---|---|---|
| | | Pre-invasive | | Micro-invasive | |
| Age group | Women screened | No. | % | No. | % |
| < 30 | 10,676 | 29 | 0·27 | 1 | 0·01 |
| 30– | 7,766 | 51 | 0·66 | 12 | 0·15 |
| 35– | 6,276 | 33 | 0·52 | 16 | 0·25 |
| 40– | 5,183 | 38 | 0·73 | 14 | 0·27 |
| 45– | 4,457 | 39 | 0·87 | 13 | 0·29 |
| 50– | 4,094 | 20 | 0·49 | 14 | 0·34 |
| 55– | 3,383 | 9 | 0·21 (55–75+) | 3 | 0·17 (55–75+) |
| 60– | 1,457 | 3 | | 3 | |
| 65– | 696 | 0 | | 3 | |
| 70– | 405 | 1 | | 2 | |
| 75+ | 318 | 0 | | 0 | |
| N.S. | 352 | 0 | 0·00 | 0 | 0·00 |
| Total : | 45,063 | 223 | 0·49 | 81 | 0·18 |

Of the 571 patients with clinical cancer, 61 per cent were aged 50 or over at the time of first attendance at hospital. The proportion varies according to the stage of the disease. The mean age of patients presenting with cancer in Stage I is 49·2 years, Stage II 51·9 years and Stages III and IV combined 56·5 years. In attempting to estimate the progression of cancer, however, it would be inadvisable to relate these mean ages to the mean ages of patients with pre-clinical cancer, for reasons discussed by Knox (1966).

Table II shows a concentration of elderly patients in the more advanced stages. In fact, staging of the disease is less accurate in the elderly because of the shrinkage of tissues, so that the vaginal vault narrows and the cervix may be completely covered over by the vaginal walls. In these circumstances, cancer of the cervix may involve the vaginal walls from the beginning. Table II shows that of the 47 cases of clinical cancer in women of 70 years or over in whom the staging was given, only 5 or 10·6 per cent were classified as Stage I. In addition, in the very old the cancer may differ in aetiology, having more of the characteristics of a slow-growing degenerative disease rather than of a direct response to a carcinogenic agent.

TABLE II.—*Age of Patients With Clinical Cancer of the Cervix, by Stage*

| | Clinical cancer | | | |
|---|---|---|---|---|
| Age group | Stage I | Stage II | Stages III and IV | Not stated |
| < 30 | 2 | 3 | 0 | 0 |
| 30 | 4 | 11 | 6 | 6 |
| 35 | 11 | 17 | 8 | 10 |
| 40 | 23 | 19 | 22 | 7 |
| 45 | 14 | 29 | 24 | 8 |
| 50 | 17 | 28 | 33 | 14 |
| 55 | 9 | 22 | 35 | 13 |
| 60 | 9 | 26 | 26 | 12 |
| 65 | 5 | 15 | 22 | 6 |
| 70 | 4 | 5 | 14 | 5 |
| 75 | 1 | 3 | 10 | 3 |
| 80 | 0 | 3 | 6 | 0 |
| 85+ | 0 | 0 | 1 | 0 |
| Total: | 99 | 181 | 207 | 84 |

Table III shows discovery rates for patients with pre-clinical cancer (pre-invasive and micro-invasive combined) by number of pregnancies (including abortions) as well as age, and (below) the numbers on which the rates are based.

Despite fluctuations attributable to small numbers, the discovery rate tends to rise to a peak at ages 45–49 in parous women with from one to four pregnancies. With five or more pregnancies quite a different pattern is seen. Rates are highest in the younger age-groups and fall with increasing age. At 50–54 and particularly at 55 and over they are low in comparison with those for younger women with the same number of pregnancies.

These findings are strikingly summarised in Fig. 1, which shows (in bars) the discovery rate of pre-clinical cancer in five age and number-of-pregnancies groups

FIG. 1.—Discovery rates of pre-clinical cancer in different age and number-of-pregnancy groups, and percentage of total women screened in each group.

TABLE III.—*Pre-Clinical Cancer Discovery Rates, by Age and Number of Pregnancies : North-East Region*, 1958–65

| Age group | Total | Number of pregnancies 0 | 1 | 2 | 3 | 4 | 5 | 6 | 7+ | N.S. |
|---|---|---|---|---|---|---|---|---|---|---|
| (a) Per 100 women screened | | | | | | | | | | |
| < 30 | 0·28 | 0·00 | 0·03 | 0·11 | 0·27 | 0·69 | 2·34 | 3·64 | 2·94 | 0·00 |
| 30– | 0·81 | 0·45 | 0·23 | 0·50 | 0·95 | 0·88 | 2·56 | 2·37 | 2·32 | 0·00 |
| 35– | 0·78 | 0·00 | 0·31 | 0·58 | 0·99 | 1·20 | 1·34 | 1·57 | 0·80 | 0·00 |
| 40– | 1·00 | 0·44 | 1·06 | 0·69 | 1·26 | 1·07 | 0·28 | 2·64 | 2·14 | 0·00 |
| 45– | 1·17 | 0·43 | 1·00 | 1·13 | 1·44 | 1·43 | 1·20 | 1·15 | 1·33 | 0·62 |
| 50– | 0·83 | 1·27 | 1·02 | 0·57 | 1·09 | 0·45 | 1·11 | 1·10 | 0·74 | 0·00 |
| 55+ | 0·38 | 0·48 | 0·16 | 0·31 | 0·72 | 0·15 | 0·44 | 0·67 | 0·66 | 0·00 |
| N.S. | 0·00 | 0·00 | 0·00 | 0·00 | 0·00 | 0·00 | 0·00 | 0·00 | 0·00 | 0·00 |
| Total: | 0·67 | 0·40 | 0·37 | 0·46 | 0·88 | 0·85 | 1·36 | 1·71 | 1·18 | 0·06 |
| (b) Women screened | | | | | | | | | | |
| < 30 | 10,676 | 431 | 3,051 | 3,652 | 1,857 | 866 | 342 | 110 | 68 | 299 |
| 30– | 7,766 | 223 | 1,292 | 2,410 | 1,792 | 1,016 | 507 | 211 | 129 | 186 |
| 35– | 6,276 | 224 | 956 | 1,716 | 1,406 | 833 | 448 | 254 | 250 | 189 |
| 40– | 5,183 | 226 | 851 | 1,457 | 1,029 | 652 | 353 | 227 | 234 | 154 |
| 45– | 4,457 | 234 | 702 | 1,242 | 830 | 558 | 332 | 174 | 225 | 160 |
| 50– | 4,094 | 236 | 782 | 1,052 | 735 | 442 | 270 | 182 | 270 | 125 |
| 55+ | 6,259 | 418 | 1,243 | 1,303 | 976 | 680 | 451 | 298 | 601 | 289 |
| N.S. | 352 | 7 | 38 | 64 | 33 | 33 | 6 | 8 | 4 | 159 |
| Total: | 45,063 | 1,999 | 8,915 | 12,896 | 8,658 | 5,080 | 2,709 | 1,464 | 1,781 | 1,561 |
| (c) With pre-clinical cancer | | | | | | | | | | |
| < 30 | 30 | 0 | 1 | 4 | 5 | 6 | 8 | 4 | 2 | 0 |
| 30– | 63 | 1 | 3 | 12 | 17 | 9 | 13 | 5 | 3 | 0 |
| 35– | 49 | 0 | 3 | 10 | 14 | 10 | 6 | 4 | 2 | 0 |
| 40– | 52 | 1 | 9 | 10 | 13 | 7 | 1 | 6 | 5 | 0 |
| 45– | 52 | 1 | 7 | 14 | 12 | 8 | 4 | 2 | 3 | 1 |
| 50– | 34 | 3 | 8 | 6 | 8 | 2 | 3 | 2 | 2 | 0 |
| 55+ | 24 | 2 | 2 | 4 | 7 | 1 | 2 | 2 | 4 | 0 |
| N.S. | 0 | 0 | 0 | 0 | 0 | 0 | 0 | 0 | 0 | 0 |
| Total: | 304 | 8 | 33 | 60 | 76 | 43 | 37 | 25 | 21 | 1 |

and (in line) each age and number-of-pregnancies group as a percentage of the total women screened (less the "not stated"). For example, the left hand side of the diagram shows that only 3 per cent of the 43,000 women screened were under age 35 and had 5 or more pregnancies, but of this group 2·6 per cent were found to have pre-clinical cancer, the highest rate in all the 5 groups. The discovery rate falls as the numbers screened increase to include women in wider age and parity groups, thus emphasising the selective nature of the disease.

Unfortunately, it has not been possible to obtain the husband's occupation for more than a small proportion of the 45,000 women screened. Women who come for cytological screening are unlikely to be a representative sample of the population and comparison of detection rates of cancer diagnosed in this way must take account of the social class and age composition of the groups concerned. Macgregor and Baird (1963) have shown that when women on the lists of general practitioners are examined the detection rate varies according to the district of the city in which the doctor's surgery is situated since this influences the social class composition of the practice, which in turn affects both the percentage of women who attend for examination and the incidence of cancer cases detected amongst those who are examined.

### *Marital Situation*

Unmarried women with pre-clinical cancer were not interviewed because of the difficulty of finding suitable controls. The marital situation of the ever-married pre-clinical group and of patients with clinical cancer who were interviewed is shown in Table IV.

TABLE IV.—*Marital State*

| | Pre-clinical | Controls | Clinical: Aberdeen | Clinical: Dundee |
|---|---|---|---|---|
| Single | 0 | 0 | 3 | 2 |
| Married once only : | | | | |
| Still married | 161 | 181 | 32 | 35 |
| Separated | 12 | 3 | 7 | 6 |
| Widowed | 8 | 5 | 12 | 9 |
| Divorced | 2 | 1 | 1 | 2 |
| Twice married, first ended by : | | | | |
| Death | 5 | 4 | 5 | 2 |
| Divorce | 12 | 6 | 2 | 2 |
| Three times married | 0 | 0 | 0 | 3 |
| Total | 200 | 200 | 62 | 61 |

Boyd and Doll (1964) found a high frequency of broken first marriages in both patients and controls (average age 52) marrying under age 20, whether due to the death of the husband, separation or divorce. With later marriage, however, significantly more patients than controls had had a broken marriage. They conclude that the high proportion of broken marriages in their patients (45 per cent compared with 29 per cent in gynaecological controls) cannot be explained wholly on the grounds of early marriage. Aberdeenshire patients with pre-clinical cancer, being younger, have had less time in which to have a broken marriage, so that a lower frequency in them is not surprising. For all ages at marriage combined, the number of broken marriages is significantly higher in patients (19·5 per cent) than in controls (9·5 per cent). In controls, the broken marriages were equally likely to have ended in widowhood as in divorce or separation. In patients, however, twice as many had ended in divorce as in widowhood.

The death of the husband is unlikely to determine the onset of cancer or to accelerate its progress by emotional influences, although the possibility has been

TABLE V.—*Broken Marriages*

| | Pre-clinical patients | | | | | Controls | | | | |
|---|---|---|---|---|---|---|---|---|---|---|
| | | Broken marriages | | | | | Broken marriages | | | |
| Age at marriage | Total | No. | % | Div. or sep. | Widowed | Total | No. | % | Div. or sep. | Widowed |
| < 20 | 51 | 11 | 21·6 | 9 | 2 | 59 | 8 | 13·5 | 5 | 3 |
| 20–24 | 109 | 24 | 22·0 | 15 | 9 | 93 | 8 | 8·6 | 4 | 4 |
| 25+ | 40 | 4 | 10·0 | 2 | 2 | 48 | 3 | 6·2 | 1 | 2 |
| Total : | 200 | 39 | 19·5 | 26 | 13 | 200 | 19 | 9·5 | 10 | 9 |

discussed. In the present study, 94 patients with clinical cancer had been widowed, and in 19 the interval from husband's death to the patient's first attendance at hospital was under 3 years, while the median interval for the whole group of 94 widows was 8 years. In view of the average length of the latent period in cervical cancer, the disease obviously must have been present for years before widowhood.

Divorce would seem to be more directly related to the circumstances which could promote cancer of the cervix than widowhood. Berggren (1957) showed the rate of cervical cancer in Sweden in 1950 to be 6·48 per 10,000 divorced women compared with 2·65 in widows. Lombard and Potter (1950) found that 20·7 per cent of patients with cervical cancer had been either separated or divorced compared with 6·7 per cent of matched controls. Separation and divorce are part of the general picture of sexual vicissitudes in the patient with cervical cancer, associated with early coitus, illegitimate pregnancies, multiple marriages and so on. Discussing their marital problems, these women did not blame incompatibility in sexual matters for their broken marriages. In general, the working class woman has a low expectation of sexual satisfaction in marriage. Rather they described troubles over money, religion, drink and other women.

### *Coitus, Marriage and Pregnancy*

All surveys show that cancer of the cervix occurs most frequently in parous married women. It is more frequent in infertile married women than in single women. Stocks (1955) found that infertile married women suffered about double the mortality from cancer of the cervix of single women of corresponding ages. Lawson's (1957) figures also suggest that marriage *per se* more than doubles the risk of cervical cancer. Squamous cell cancer of the cervix occurs rarely, if ever, in virgins, although in them adenocarcinoma is less unusual.

The stimulus is therefore presumably coitus and not pregnancy. It is assumed that risk starts at the age of first coitus which in many cases coincides with marriage. Many studies have shown early marriage to be more common in patients than in controls. The Aberdeenshire epidemiological data, however, described by Aitken-Swan and Baird (1966), showed no significant difference between observed and expected numbers of patients marrying under age 20 when the population was standardised to the composition of the patients for age, number of live-born children and husband's socio-economic group simultaneously. This finding can now be checked in the group with pre-clinical cancer and controls matched for age and number of pregnancies. Whether divided into pre-invasive and micro-invasive cancers, city or county residence or all 200 considered together, there is no excess of patients marrying under age 20 (Table VI).

TABLE VI.—*Age at Marriage : Patients with Pre-Clinical Cancer and Controls*

| Age at marriage | Total | | Pre-invasive | | Micro-invasive | | City | | County | |
|---|---|---|---|---|---|---|---|---|---|---|
| | Patients | Controls | Patients | Controls | Patients | Controls | Patients | Controls | Patients | Controls |
| <20 . | 51 | 59 | 39 | 42 | 12 | 17 | 39 | 39 | 12 | 20 |
| 20–24 . | 109 | 93 | 80 | 67 | 29 | 26 | 81 | 72 | 28 | 21 |
| 25+ . | 40 | 48 | 25 | 35 | 15 | 13 | 23 | 32 | 17 | 16 |
| Total: . | 200 | 200 | 144 | 144 | 56 | 56 | 143 | 143 | 57 | 57 |

It is surprising that more controls than patients married under age 20 since the lower socio-economic groups, in whom early marriage is more common, predominate among patients with cervical cancer. Would the same effect be found if controls were matched for socio-economic group as well as age, parity and source of contact? Sixty-two pairs happen to be similar in all four respects. Table VII shows clearly that there is no difference between these particular patients with cervical cancer and controls in their age at marriage.

TABLE VII.—*Age at Marriage of Patients with Pre-Clinical Cancer and Controls Matched for Age, Number of Pregnancies, Source of Contact and Socio-Economic Group*

| Age at marriage | All areas | | City | | County | |
|---|---|---|---|---|---|---|
| | Patients | Controls | Patients | Controls | Patients | Controls |
| < 20 | 19 | 19 | 15 | 15 | 4 | 4 |
| 20– | 32 | 30 | 26 | 24 | 6 | 6 |
| 25– | 9 | 10 | 5 | 7 | 4 | 3 |
| 30+ | 2 | 3 | 2 | 2 | 0 | 1 |
| Total: | 62 | 62 | 48 | 48 | 14 | 14 |

In view of the consistent findings of the world literature that patients with cervical cancer are younger at marriage than controls, one might hestitate to accept the figures in Tables VI and VII as more than a chance result arising from small numbers were it not for two considerations. First, the tables support the epidemiological findings described in the preceding paper, showing no significant excess of patients marrying under age 20. Second, the 200 patients with pre-clinical cancer and controls in Tables VI and VII are more closely matched to each other than is the case in other studies. Table VII suggests that very close matching for age, number of pregnancies, source of contact and socio-economic group may obliterate the difference in age at marriage between patients with cancer of the cervix and controls.

The question of how adequately age at marriage represents age at first coitus is relevant to the further study of these results. In 114 of the pre-clinical cases in the city age at first coitus was obtained for both patient and control. Table VIII shows these 228 cases divided into 5 socio-economic groups and the proportion in each group where the stated age at first coitus was lower than age at marriage. These results are also given for patients and controls separately.

TABLE VIII.—*Pre-Marital Coitus ; Patients with Pre-Clinical Cancer and Controls, City*

| Socio-economic group | Patients and controls combined | | | Patients | | Controls | |
|---|---|---|---|---|---|---|---|
| | Total cases | Pre-marital coitus | % | Total patients | Pre-marital coitus | Total controls | Pre-marita coitus |
| Professional managerial | 24 | 2 | 8·3 | 6 | 0 | 18 | 2 |
| Intermediate non-manual | 23 | 6 | 26·1 | 11 | 3 | 12 | 3 |
| Skilled manual workers | 95 | 34 | 35·8 | 47 | 23 | 48 | 11 |
| Semi-skilled workers | 50 | 17 | 34·0 | 27 | 11 | 23 | 6 |
| Unskilled workers | 36 | 17 | 47·2 | 23 | 12 | 13 | 5 |
| Total: | 228 | 76 | 33·5 | 114 | 49 | 114 | 27 |

In a third of the combined patients and controls age at first coitus is not the same as age at marriage; this proportion varies from 8·3 per cent in the highest socio-economic group to almost half in the lowest. As will be seen later, pre-marital coitus in women in the non-manual groups may be understated. All the figures obviously represent a minimum. It is clear, therefore, that age at marriage becomes a less reliable indicator of age at first coitus as social class declines, although the use of 5-year categories (under 20, 20–24, etc.) means that age at first coitus and age at marriage may still fall into the same 5-year age group.

More striking still is the difference between patients and controls in the proportion who have had a completed pregnancy or pregnancies before marriage to the same or another man (Table IX). Here pregnancies include abortions and only those completed before marriage are included. Three times as many patients with pre-clinical cancer as controls had had such a pregnancy. The high rate in patients is confirmed in the group with clinical cancer.

TABLE IX.—*Pre-Marital Pregnancy in Patients with Pre-Clinical Cancer and Controls, and Clinical Cancer : City and County*

| | Controls for pre-clinical | | | Pre-clinical | | | Clinical | | |
|---|---|---|---|---|---|---|---|---|---|
| Socio-economic group | Total controls | Pre-mar. preg. | % | Total patients | Pre-mar. preg. | % | Total patients | Pre-mar. preg. | % |
| Professional, managerial | 27 | 0 | 0·0 | 7 | 0 | 0·0 | 34 | 2 | 5·9 |
| Intermediate non-manual | 24 | 0 | 0·0 | 20 | 1 | 5·0 | 36 | 4 | 11·1 |
| Skilled manual workers | 72 | 7 | 9·7 | 68 | 16 | 23·5 | 151 | 28 | 18·7 |
| Semi-skilled workers | 40 | 4 | 10·0 | 50 | 6 | 12·0 | 116 | 23 | 20·0 |
| Unskilled workers | 21 | 2 | 9·5 | 29 | 9 | 31·0 | 86 | 21 | 24·4 |
| Farmers, farm workers | 16 | 1 | 6·2 | 26 | 10 | 38·5 | 45 | 13 | 28·9 |
| Not stated | 0 | 0 | 0·0 | 0 | 0 | 0·0 | 15 | 4 | — |
| Total : | 200 | 14 | 7·0 | 200 | 42 | 21·3 | 483* | 95 | 19·8 |

* 50 omitted where age at marriage or date of first pregnancy not stated.

The patients with pre-clinical cancer and their controls in Table IX are women who accepted an invitation to take part in the cytological screening and to this extent they all come from the same " population ". While patients accounted for two-thirds of the combined patients and controls who had pre-marital coitus (49 out of 76, Table VIII), they account for three-quarters of the number who had a pre-marital pregnancy (42 out of 56, Table IX). The difference between patients and controls in the occurrence of these events is highly significant and is seen in all but one occupational class, the professional and managerial (Table VIII) where numbers are particularly small.

The proportion of women who had a pre-marital pregnancy rises steeply as age at marriage rises (Table X). This is so in each occupational class separately, although of course in the non-manual group the rate of pre-marital pregnancies is much lower than in the other groups.

Table X shows that as an indication of age at the start of exposure to the supposed carcinogenic stimulus the age at marriage category " 25 and over " is the least reliable, with nearly a third of the women having had pregnancies or coitus before that age. The delay in marriage suggests that in many of these cases the man married was not the father of the child.

TABLE X.—*Per Cent of Patients with Clinical Cancer in Each Age at Marriage and Socio-Economic Group Who Had Had a Pre-Marital Pregnancy*

| | Percentage of clinical patients who had had a pre-m. pregnancy | | | Number of clinical patients | | |
|---|---|---|---|---|---|---|
| | Age at marriage | | | Age at marriage | | |
| | < 20 | 20–24 | 25+ | < 20 | 20–24 | 25+ |
| Socio-economic group | % | % | % | No. | No. | No. |
| Non-manual workers | 0 | 8 | 11 | 7 | 39 | 27 |
| Skilled manual workers | 3 | 16 | 30 | 33 | 71 | 54 |
| Semi-skilled manual | 5 | 20 | 36 | 37 | 50 | 31 |
| Unskilled workers | 6 | 29 | 37 | 31 | 34 | 24 |
| Farmers, farm workers | 17 | 25 | 37 | 6 | 20 | 19 |
| Total: | 6 | 19 | 30 | 114 | 214 | 155 |

Tables VIII and IX have shown that in all the occupational groups except the non-manual, pre-marital coitus is more common in patients than controls and that more patients than controls had had a pre-marital pregnancy (both significant at 0·5 per cent level). This being so, it is not surprising that age at first coitus is earlier in patients than in controls. Age at first coitus and its relation to age at marriage in the same group can be compared in 40 patients with pre-clinical cancer who are matched for age, number of pregnancies, and source of contact and, particularly important, who are in the same socio-economic group as the controls (Table XI).

TABLE XI.—*Age at First Coitus and Age at Marriage of Patients With Pre-Clinical Cancer and Controls Matched for Age, Number of Pregnancies, Source of Contact and Socio-Economic Group*

| | First coitus | | Marriage | |
|---|---|---|---|---|
| Age | Patients | Controls | Patients | Controls |
| < 20 | 25 | 15 | 15 | 12 |
| 20–24 | 12 | 18 | 19 | 19 |
| 25–29 | 2 | 6 | 4 | 7 |
| 30+ | 1 | 1 | 2 | 2 |
| Total: | 40 | 40 | 40 | 40 |

From all the evidence presented in Tables VI–XI, it would appear that what specially distinguishes the patient with pre-clinical cancer from the controls is not earlier marriage so much as the tendency to have coitus and pregnancies before marriage, which in turn helps to define the section of the population from which patients are drawn. This emphasises again the need for the most careful matching of socio-economic background in any comparisons involving sexual behaviour.

*Interval from First Coitus or Marriage to First Attendance at Hospital*

Tables VIII and IX have shown that in many cases age at marriage differs from age at first coitus. Age at first coitus is not recorded in the medical case-notes and is only available for patients and controls who were specially interviewed for the study. The interval between first coitus and the diagnosis of cancer cannot therefore be stated precisely for the whole series. The interval is calculated from the age at first coitus, where available, or marriage or first pregnancy, whichever was the earlier. This at least reduces the margin of error.

The median interval from this first known stimulus to first attendance at hospital is not affected by whether the first stimulus occurred under age 20 or at 20–24, being 31 years in both cases. When it was said to be at 25 or over, the interval was shorter (25 years). This could be because in fact first coitus occurred before age 25 in many cases. That this is so is suggested by a comparison of the proportion in the three categories (first stimulus under age 20, 20–24 or 25 and over) who attended hospital in *less than* 10 *years* from first stimulus, an unusually short interval for the development of clinical cancer of the cervix. This is 0·6 and 1·8 per cent in those whose first stimulus was under age 20 and 20–24 respectively, but 6·3 per cent of those in whom the first stimulus was stated to be 25 or over.

The median interval between first stimulus and first attendance at hospital with clinical cancer is almost the same in 3 broad occupational classes, non-manual (29 years), skilled manual (31 years) and unskilled manual workers (30 years). The protection that the background and way of life of the non-manual classes affords seems to be more in lessening the incidence of the disease than in lengthening the time taken for the cancer to progress through its various stages in those who actually develop it. Again there is an interesting occupational class difference in the percentage of patients attending in less than 10 years after the alleged time of first stimulus—1 per cent in the skilled manual, 2 per cent in the unskilled manual and 7 per cent in the non-manual workers, suggesting that women in the non-manual group are less willing than others to divulge pre-marital coitus.

Nothing stands out in the social data to account for the great range of time intervals. Although some women who had clinical cancer before age 35 claimed to be happily married and to have had one partner, others had more marital vicissitudes than usual, numerous partners, illegitimate children and husbands who drank to excess or had been in prison, but similar situations also occurred when the interval was longer. What impresses about the short interval cases is the concentration of unusual events in the lives of such young women, for example :

> First coitus said to be just before marriage at 21. Three marriages and 6 pregnancies by the age of 33 when she first attended with clinical cancer of the cervix (Stage I).
>
> First coitus at 15. Two illegitimate children before marriage at 20. Divorced within 3 years. Cancer of the cervix at age 26 (Stage II*a*).
>
> Married at 18. Three children in 3 years, then widowed. Three more in the following 4 years, all to different men. Clinical cancer of the cervix at age 26 (Stage I).

The only other patient in the series who had clinical cancer as early as 26 years of age was described as a " social problem ", was separated from her husband and had one illegitimate child. The two youngest patients with *pre-invasive* cancer were aged 24, having married at 17 and 19 respectively. One had 5 children and the other 6 ; in both the diagnosis was made when they entered hospital for termination of pregnancy by hysterotomy and tubal ligation because of extreme debility and adverse environmental factors.

However, rapid child-bearing and high parity in young women is not enough in itself to account for the short interval between the start of coitus and the development of cancer of the cervix. Other patients who had had 5 or 6 children in rapid succession did not develop clinical cervical cancer until they were middle-aged, for example :

> This patient had 7 pregnancies (including a still-birth and an abortion) in 6 years, and went on to have another 2 in the next 8 years before attending hospital with cancer of the cervix (Stage II) at age 59. She had married over the age of 25 and claimed that coitus had not occurred before then. Her husband was found on physical examination to be fully circumcised.
>
> Another patient married at age 19 (first coitus at 19) had 7 pregnancies in 6 years (including one abortion), had a further 2 during the next 6 years and first attended hospital with cancer of the cervix (Stage II*b*) at age 52.

There are fewer illegitimate births and less mention of " social problems " and partners other than the husband in the medical histories of these women who, although they had numerous pregnancies in rapid succession, did not develop cancer until comparatively late in life. One is left with the strong impression that sexual promiscuity could be an important predisposing factor in the development of clinical cancer at a very early age.

Child-bearing increases the risk of cervical cancer, but childless patients do not appear to get the disease any later in life than those who have borne children. A total of 5 patients with pre-clinical cancer and 30 ever-married patients with clinical cancer in Aberdeenshire and Dundee combined had had no live-born children, of whom 3 and 23 respectively said they had had no pregnancies at all. The proportion of childless marriages is therefore 2·5 and 4·6 per cent of all marriages in the pre-clinical and clinical groups, and of totally sterile marriages 1·5 and 3·7 per cent respectively. The average age at first attendance both of childless patients and of those who had borne children closely approximates 52·5 years. Of particular interest is the interval in the patients who have had no pregnancies at all. In one of the 3 with *pre-clinical* cancer of the cervix preinvasive cancer was found at age 48, 23 years after first coitus ; in a second micro-invasive cancer was found at age 40, 21 years after her first marriage and 12 years after a second marriage. In the third patient, micro-invasive cancer was found at age 53, 22 years after marriage. These intervals are very similar to the intervals for all patients with pre-clinical cancer regardless of child-bearing history.

Three-quarters of the patients with *clinical* cancer who had never been pregnant were aged 25 or over at marriage. As already stated, many women marrying later in life started coitus years before marriage, so that the interval in this group cannot be worked out with any accuracy. It is interesting to note, however, that 3 patients who had never been pregnant first attended with clinical cancer as early as ages 32, 33 and 34, all Stage II. The interval from first known stimulus (marriage) to first attendance at hospital was 5, 9 and 8 years respectively, but theoretically it could have been as long as 17–19 years. Even so, this interval to Stage II clinical cancer is very short, particularly in women who have never been pregnant, and may be associated with adverse social factors. Unfortunately, they were not interviewed for the study but in one case (attending at age 32) the patient was known to have been treated for venereal disease at age 23.

### *Circumcision*

Whether male circumcision protects the cervix from the risk of cancer is not yet known. Comparison of the incidence in different ethnic groups who practice and do not practice circumcision suggests that it does, but when husbands of patients and controls in more homogeneous populations are compared there is less evidence of an association. A recent study (Aitken-Swan and Baird, 1965), based on the physical examination of husbands of women interviewed for the present inquiry, found no significant difference in length of foreskin between husbands of women with and without cancer of the cervix.

Not all the husbands whose wives were interviewed co-operated in that study. However, most wives were asked routinely if their husbands had been circumcised. In those cases where the husband was examined by a doctor, comparison of the wife's statement with the doctor's opinion on whether or not surgical circumcision had been done, showed that when wives were *certain* they knew they were in fact right in almost every case. When they only *thought* they knew the error was greater.

Table XII makes use of all the data available to show the surgical circumcision status of husbands of patients and controls, as determined by physical examination or by the wife's positive statement. The other categories are also shown.

TABLE XII.—*Circumcision Status of Husbands*

| | Controls | | Patients | | | |
|---|---|---|---|---|---|---|
| | | | Total | | Pre-clin. | Clinical |
| *Circumcised :* | | | | | | |
| Seen by doctor | 13 | | 11 | | 8 | 3 |
| Not seen by doctor but wife *sure* he is circ. | 5 | | 12 | | 7 | 5 |
| TOTAL : | 18 | 23·4% | 23 | 21·1% | 15 | 8 |
| *Not circumcised :* | | | | | | |
| Seen by doctor | 37 | | 40 | | 34 | 6 |
| Not seen by doctor : wife *sure* he is not circ. | 22 | | 46 | | 21 | 25 |
| TOTAL : | 59 | 76·6% | 86 | 78·9% | 55 | 31 |
| *Uncertain :* | | | | | | |
| Seen by doctor | 6 | | 3 | | 2 | 1 |
| Not seen by doctor : | | | | | | |
| Wife *thinks* he is circumcised | 4 | | 3 | | 3 | 0 |
| Wife *thinks* he is not circumcised | 33 | | 36 | | 18 | 18 |
| Wife does not know | 25 | | 77 | | 32 | 45 |
| Not asked | 6 | | 35 | | 18 | 17 |
| TOTAL : | 74 | | 154 | | 73 | 81 |

In the case of 77 controls and 109 patients, the doctor had a definite opinion or the wife was sure she knew if her husband was circumcised or not. Of these, 23·4 per cent of husbands of controls and 21·1 per cent of patients' husbands were probably circumcised. The inclusion of the wife's positive statement does not therefore alter the conclusion reached in the earlier study, that there is no significant difference between the circumcision status of husbands of patients and controls.

*Contraception*

From the apparent association between coitus, sexual hygiene and cancer of the cervix, it would seem to follow that obstructive methods of contraception should have a protective effect against cancer and that the regular use of a sheath or cap should be less common among patients and their husbands than among controls. The study by Wynder *et al.* (1954) indicated that more patients than controls had not practised any form of contraception and that there was a slightly greater use of sheath contraception by husbands of controls than by husbands of patients, although the frequency of use was not obtained. Boyd and Doll (1964) also found the use of obstructive methods to be more frequent in their control group than in patients ; on the other hand, no such difference was found in the studies of Jones, Macdonald and Breslow (1958) or Lombard and Potter (1950). The contradictory evidence may be partly due to the difficulty of getting reliable information and of defining such terms as " regular use ". The period of married life at which the methods were used may also be relevant but this is not easy to recall or to summarise. Only the broadest comparison can be made here. Patients and controls were asked if they had ever used specified methods, how long and how regularly they had used them and if any method had been predominantly used (Table XIII).

TABLE XIII.—*Per Cent Distribution of Use of Sheath or Cap Contraception : Patients With Clinical Cancer Under Age 50, With Pre-Clinical Cancer and Controls*

| | | | | Occupational group | | | | | |
|---|---|---|---|---|---|---|---|---|---|
| | | | | Non-manual | | Skilled manual | | Unskilled manual | |
| Use of sheath or cap contraception | Mean age (years) | Patients % | Controls % | Patients % | Controls % | Patients % | Controls % | Patients % | Controls % |
| Regular use | 35 | 12 | 20 | 8 | 30 | 15 | 20 | 10 | 13 |
| Occasional use | 37 | 32 | 46 | 50 | 54 | 44 | 40 | 17 | 48 |
| Not used at all | 42 | 51 | 34 | 38 | 16 | 38 | 40 | 66 | 39 |
| Not stated | — | 5 | 0 | 4 | 0 | 3 | 0 | 7 | 0 |
| Total : | — | 100 | 100 | 100 | 100 | 100 | 100 | 100 | 100 |
| | | (199) | (143) | (24) | (37) | (81) | (58) | (94) | (48) |

Note : Actual numbers in parentheses.

The use of contraceptives and the type used differ in people of different ages and occupational groups. In the present study, the patients with pre-clinical cancer and controls are matched for age. Patients with clinical cancer are generally older and many were producing their families before appliance methods of birth control came into general use. To take account of this, only patients under age 50 with clinical cancer are included in the comparison. Results might also be affected by the preponderance among patients of the lower socio-economic groups, fewer of whom use mechanical methods. Table XIII compares the use of sheath or cap in 3 occupational groups separately, although numbers become very small. Taken as a whole, more controls (20 per cent) than patients (12 per cent) said they had " regularly " used a sheath or cap as their predominant method, but the difference is barely significant.

*Discussion*

The cytological screening of 45,000 women in this region in 6 years has resulted in finding 304 pre-clinical cancers of the cervix, a rate of 0·7 per cent. Two hundred and twenty-three were in the pre-invasive stage and 81 were already invading the tissues below the epithelium though presenting no symptoms. The highest incidence of pre-clinical cancers was found in women aged under 35 who had had 5 or more pregnancies (Table III). Their rate was $3\frac{1}{2}$ times above the average (2·56 per cent compared with 0·7 per cent). Those with 5 or more pregnancies but over the age of 35 did not have the same high rates as the younger women. High parity in young women tends to be associated with certain social characteristics such as early marriage, pre-marital conception, short stature, high post-neonatal mortality rates, often allied to poor physical health, sub-standard living conditions and poor hygiene. While there may be no direct causal relationship between cancer of the cervix and too rapid and frequent child-bearing, these occurrences in women who have a predisposition to cancer may activate the tendency and accelerate its development. Some particular susceptibility would appear to be involved, since even in such women the discovery rate of pre-clinical cancer of the cervix is less than 4 per cent of those screened. On the other hand, it may be that associated aspects of their lives lead to cancer in women with this reproductive pattern, for example, intercourse of unusual frequency, lack of protection by sheath contraception, poor penile hygiene in their partner, intercourse with a number of partners, or any combination of such factors operative at an early age.

Discovery rates by the use of cytology fall off surprisingly quickly after the age of 55. Possibly, women who have a particular tendency to cancer develop the disease in the younger age groups and are cured or die before they reach 55 or 60 in most cases. Again, women who might have cancer, or who feared they had cancer, might decline to participate in screening ; these might be mainly older women. The number of such cases is probably too small to affect the total rate, but might be enough to affect the reliability of a rate in an age and parity sub-group.

From the practical point of view, if resources are not sufficient to offer a cytological screening service to all women irrespective of differences in risk, the first priority should clearly be younger women of high parity. Paradoxically, it is often difficult to persuade these high risk groups to come for screening and the opportunity of contact with them in the post-natal period should not be lost. A second priority should be all women who have had three or more pregnancies, since the discovery rate nearly doubles at this point.

Among the complex environmental influences in cancer of the cervix, it appears likely that individual sexual habits and hygiene play a major aetiological role. The wide variation in the incidence of cervical cancer in different population groups could be largely a reflection of differences in their sexual habits and hygiene. The high incidence in Denmark (Fig. 1, Aitken-Swan and Baird, 1966) is probably not unrelated to its high abortion and divorce rates. The distribution curves for Aberdeenshire, Liverpool and Birmingham, which are not unlike each other, may give a picture characteristic of a British pattern of sexual behaviour.

It has been shown (Aitken-Swan and Baird, 1966) that there is a steep social class gradient in the incidence of cancer of the cervix, but one of the most interesting findings of the present paper is the difference in sexual behaviour of women with pre-clinical cancer (and also clinical cancer, as they follow the same pattern) and controls, even when matched for socio-economic group. The cancer patient is characterised by more marital misadventures, divorce and separation, more pre-marital coitus and deliveries and more sexual partners. Since only a very small percentage of those who have coitus frequently with partners whose penile hygiene is poor or who do not use obstructive methods of contraception actually get cancer, one must postulate that either relatively few women are susceptible to the carcinogenic stimulus or that very few of their partners do in fact carry it. In the latter case, obviously the greater the number of partners the greater the chance of exposure to the carcinogen.

If the pattern of sexual behaviour just described creates circumstances favourable to the development of cervical cancer in susceptible individuals, what of the prostitute who, as Røjel (1953) points out, may have coitus with different partners up to 10 or 15 times in 24 hours? In theory, this should produce a very high ratio of prostitutes to other women in any group of younger patients with cervical cancer. In Røjel's group, their number among patients was about four times that among controls of the same social level, or five times if the category is extended to include semi-prostitutes. That it is not higher may be due to the measures of sexual hygiene adopted by the prostitute which, in her own interests, may be more thorough and effective than those of other women of the same social level. The use of sheath contraception, on which many of the more professional-type prostitutes insist, gives protection against poor penile hygiene and infections which the ordinary married woman whose partners did not regularly use such methods would not have. By no means all are careful, as the high proportion of prostitutes among patients with cervical cancer shows. A medical social worker with long experience of patients with venereal disease is of the opinion that it is the poorer type of prostitute who seems to be singularly haphazard about such matters, being either young and inexperienced or older and often past the menopause (Johns, 1965, personal communication). It may be from this section that the bulk of patients with cervical cancer who are prostitutes come.

The implications of the present study can be summed up as follows :

1. Before cancer of the cervix can develop it would seem that there must be a pre-existing susceptibility to the disease. This may be influenced by racial, genetic, nutritional, hormonal or other factors, or by a combination of such factors.

2. In the few (among the unknown number of susceptible individuals) who actually develop the disease, it would appear to be some attribute of coitus which leads to the production of malignant change. Probably the frequency and duration of coitus is of less aetiological importance than the standard of hygiene, more particularly penile hygiene, and the number of partners to whom the woman is exposed.

3. There are indications that the regular use of obstructive methods of contraception and careful attention to sexual hygiene can protect against the carcinogenic stimulus in spite of behaviour conducive to the greatest risk.

4. It is unlikely that coitus in adolescence in itself predisposes to higher risk of cervical cancer through some special susceptibility at a period of physiological change. There is much evidence that the outcome of a first pregnancy is most successful about the age of 18. It seems improbable, therefore, that the cervix would be in a state of biological immaturity at this time. While it is true that marriage or first coitus under age 20 is generally found to be more common in patients than in controls of the same age, it still has to be shown that this is so for women who are also closely matched for socio-economic level and mode of life.

5. Child-bearing is not directly responsible for the development of cervical cancer, even in susceptible women, nor does it determine at what age it will appear. Only some of the patients who had had numerous pregnancies in rapid succession developed the disease early in life and in them other possibly pre-disposing circumstances were present, e.g. several partners. The excess of large families among patients with cervical cancer could be due to the fact that there is a close correlation between family size and social characteristics and patterns of living, and that when family size reaches 5 or more, and particularly 7 or more, we may be dealing with a very selected group of people. Their sexual habits and general way of life rather than the associated factor of family size are probably of the most aetiological significance.

6. Finally, cancer of the cervix is a disease of multiple causality. The type associated with coitus has characteristically a time interval of 30 years from first coitus to first attendance at hospital. Thus it occurs typically in middle age although, perhaps because aggravating circumstances are severe, it may appear very much earlier. It is possible that the carcinogenic agent could be a virus. Education to stress the importance of penile hygiene as a prophylactic would be justified but unfortunately, as in so many other situations, those most in need of the advice would be the most difficult to educate.

Cancer of the cervix sometimes appears in elderly women as long as 50 years or more after the start of coitus so that it becomes difficult to postulate first coitus as the important predisposing factor. However, it may not always be the first partner who carries the carcinogenic agent. On the other hand, in the elderly this could be a degenerative type of cancer which like many other cancers increase in frequency with advancing age. This theory would agree with Fig. 1 of Aitken-Swan and Baird (1966), which shows that despite the variation in incidence of cancer of the cervix in the younger age groups in Aberdeenshire, Liverpool and Birmingham, and particularly Denmark, the difference in incidence in the older age groups is much less. Again, if it is true that the average age of women with cervical cancer is rising, as suggested by the Registrar-General's figures quoted in the preceding paper, this may indicate that the type associated with coitus is becoming less common, perhaps not so much as a result of any changes in sexual behaviour but because the benefits of higher living standards and better hygiene are becoming more widely available than ever before.

Progress in research in this field may lie in making studies in some depth, for instance, of sexual habits, of patients with cervical cancer and carefully matched controls. In our experience, women are willing to discuss these matters, if tactfully approached. It is necessary to ensure that patients and controls come from the same section of the same social class. The use of the husband's present occupation to determine social class is convenient, and indeed essential if census populations are to be used as controls. Although women thus classified have certain broad characteristics in common, the survey has clearly shown the need for a more refined measure of socio-economic status than those used so far in studies of cervical cancer.

In a defined area such as Aberdeenshire, which is neither very large nor densely populated, there are not enough cases of cancer of the cervix for detailed analysis unless the study is retrospective or goes on for many years. The growth of cytological screening services affords the opportunity to collect information systematically from large numbers of women with pre-clinical cancer and carefully matched controls. The recording of the basic information required to enable the matching to be done would add to the work of those running the services, but every encouragement, financial and other, should be given them. Here is an opportunity to ask every woman who is screened to contribute to knowledge about cancer and to do so in the context of preventive work, which draws attention to the more hopeful aspects of cancer treatment.

### SUMMARY

Among 45,000 women in North-East Scotland screened by cervical cytology between 1958–65, the discovery rate of pre-clinical cancer of the cervix is 0·7 per cent. Women with 5 or more pregnancies, especially those under age 35, are the most at risk.

Aetiological factors are studied in 200 patients with pre-clinical cancer and matched controls. Striking differences in sexual behaviour are apparent. Patients with pre-clinical cancer resemble patients with clinical cancer and differ greatly from controls in the proportion of women who have had marital misadventures, pre-marital coitus and pre-marital pregnancies. These events are closely associated with socio-economic status, and the study emphasises the need for a more precise definition of this factor.

Cancer of the cervix, possibly of two aetiological types, are discussed ; one, stimulated by coitus and having an average latent period of 30 years and possibly controllable by improved sexual hygiene ; the other much less frequent, and possibly a degenerative disease, in which advancing age is the pre-disposing factor.

We are greatly indebted to Professor Raymond Illsley for his help in this study. We would also like to thank Dr. R. Doll, Dr. J. A. H. Waterhouse of Birmingham and Dr. M. A. Stewart of Liverpool for making available to us the incidence rates for those cities, and Professor James Walker for enabling us to interview patients in Dundee. Dr. J. Elizabeth Macgregor kindly provided the material for the analysis of the cytological data and Mr. W. Bytheway helped with statistical advice.

### REFERENCES

AITKEN-SWAN, J. and BAIRD, D.—(1966) *Br. J. Cancer*, **20**, 624—(1965) *Br. J. Cancer*, **19**, 217.

BERGGREN, O. G. A.—(1957) Acta radiol. Supp. 145, i.

BOYD, J. T. and DOLL, R.—(1964) *Br. J. Cancer*, **18**, 419.

JONES, E. G., MACDONALD, I. and BRESLOW, L.—(1958) *Am. J. Obstet. Gynec.*, **76**, 1.
KNOX, E. G.—(1966) In " Problems and Progress in Medical Care " (Second Series). London (Oxford University Press for Nuffield Provincial Hospitals Trust).
LAWSON, J. G.—(1957) *J. Obstet. Gynaec. Br. Commonw.*, **64**, 4, 488.
LOMBARD, H. L. and POTTER, E. A.—(1950) *Cancer, N.Y.*, **3**, 960.
MACGREGOR, J. E. and BAIRD, D.—(1963) *Br. med. J.*, i, 1631.
ROJEL, J.—(1953) *Acta path. microbiol. scand.*, Suppl. 97, i.
STOCKS, P.—(1955) *Br. J. Cancer*. **9**, 487.
WYNDER, E. L., CORNFIELD, J., SCHROFF, P. D. and DORAISWAMI, K. R.—(1954) *Am. J. Obstet. Gynec.*, **68**, 1016.